# Interaction effects between weather and space use on harvesting effort and patterns in red deer

**DOI:** 10.1002/ece3.1318

**Published:** 2014-12-03

**Authors:** Inger M Rivrud, Erling L Meisingset, Leif E Loe, Atle Mysterud

**Affiliations:** 1 Centre for Ecological and Evolutionary Synthesis (CEES), Department of Biosciences, University of OsloP.O. Box 1066 Blindern, NO-0316, Oslo, Norway; 2 Norwegian Institute for Agricultural and Environmental ResearchTingvoll gard, NO-6630, Tingvoll, Norway; 3 The Department of Ecology and Natural Resource Management, Norwegian University of Life SciencesPO Box 5003, NO-1432, Aas, Norway

**Keywords:** Behavior, climate, harvesting patterns, large herbivores, management, ungulates

## Abstract

Most cervid populations in Europe and North America are managed through selective harvesting, often with age- and sex-specific quotas, with a large influence on the population growth rate. Less well understood is how prevailing weather affects harvesting selectivity and off-take indirectly through changes in individual animal and hunter behavior. The behavior and movement patterns of hunters and their prey are expected to be influenced by weather conditions. Furthermore, habitat characteristics like habitat openness are also known to affect movement patterns and harvesting vulnerability, but how much such processes affect harvest composition has not been quantified. We use harvest data from red deer (*Cervus elaphus*) to investigate how weather and habitat characteristics affect behavioral decisions of red deer and their hunters throughout the hunting season. More specifically, we look at how sex and age class, temperature, precipitation, moon phase, and day of week affect the probability of being harvested on farmland (open habitat), hunter effort, and the overall harvest numbers. Moon phase and day of week were the strongest predictors of hunter effort and harvest numbers, with higher effort during full moon and weekends, and higher numbers during full moon. In general, the effect of fall weather conditions and habitat characteristics on harvest effort and numbers varied through the season. Yearlings showed the highest variation in the probability of being harvested on farmland through the season, but there was no effect of sex. Our study is among the first to highlight that weather may affect harvesting patterns and off-take indirectly through animal and hunter behavior, but the interaction effects of weather and space use on hunter behavior are complicated, and seem less important than hunter preference and quotas in determining hunter selection and harvest off-take. The consideration of hunter behavior is therefore key when forming management rules for sustainable harvesting.

## Introduction

Most cervid populations in Europe and North America are managed through some form of selective harvesting, and the way this is implemented has a huge impact on population growth rate (Solberg et al. 1999; Milner et al. 2006). Most of the selectivity arises due to management regulations such as age- and sex-specific quotas (review in Mysterud 2011), but hunter preferences also play a role, especially for trophy hunting (Coltman et al. 2003; Martinez et al. 2005; Monteith et al. 2013). Recently, it has been highlighted that harvesting selectivity may arise indirectly through animal behavior, for instance if animals make themselves more or less prone to harvest by the use of open habitat (Solberg et al. 2010; Ciuti et al. 2012). Animals of certain age and sex classes are expected to behave differently, causing differing spatial distribution and movement patterns, and thus variation in their associated probability of being harvested. Younger individuals are known to be more naïve than older, more experienced individuals (Ciuti et al. 2012), which can lead to more exposure in open habitats and thus higher harvesting risk than for older individuals. Energy requirements also differ between age and sex classes. In female mammals, lactation causes a major increase in their energetic requirements (Hanwell and Peaker 1977). Also, female cervids raising male calves have been shown to have higher energy requirements than females raising female calves (Clutton-Brock et al. 1988). Open habitats can, with forage of higher quality and quantity than in covered habitats (Albon and Langvatn 1992), attract females with higher energy requirements, but exposure in open habitats may also lead to increased harvesting vulnerability. Knowledge about typical movement patterns and behavior for the various categories, whether this affects hunter success and animal vulnerability and how this may affect harvesting selectivity indirectly is largely absent (Bunnefeld et al. 2009).

Weather conditions can affect behavior, influencing both small and large scale movement of deer (i.e., Parker and Robbins 1985; Fieberg et al. 2008) and thus affecting their likelihood of being targeted for harvest. Open habitat such as farmland is more exposed to harsh weather. During periods of heavy precipitation or low temperatures, cervids have been shown to minimize exposure to bad weather by seeking cover in the forest and thus utilize open habitat to a lesser extent (Parker et al. 1984; Mysterud and Østbye 1999). This lowers the risk of being harvested, as the animals are less exposed. Weather may also affect the behavior of the hunter (Curtis 1971), but as far as we are aware, there is no study on how human hunter effort is affected by the prevailing weather. Several environmental features, including habitat characteristics and climatic variations, are well known to affect predator–prey dynamics in general (i.e., increase in snow leading to larger pack sizes of wolves (*Canis lupus*), and a higher number of moose (*Alces alces*) killed; Post et al. 1999; Kunkel and Pletscher 2000; Lebel et al. 2012). In a human harvesting setting, hunters can be viewed as predators and the hunted animal as prey (Nugent and Choquenot 2004). Environmental variations are thus expected to influence the hunters themselves, such as extreme cold or heavy precipitation perhaps constituting less attractive hunting weather than warmer and drier conditions, but we lack quantitative information on how important weather is for hunter effort, and how this affects the harvest.

Moonlight has also been shown to affect hunting conditions for wolves, with increased hunting success during moonlit nights (Theuerkauf et al. 2003). A full moon provides brighter conditions, which can increase the harvesting risk, and more so in open habitats. For hunters, moonlight can provide attractive hunting conditions with increased visibility and longer nights, potentially increasing the hunter effort where moonlight hunting is allowed. Finally, off-road human activity has been found to be higher during weekends (Ciuti et al. 2012). The day of the week may therefore affect hunter effort and harvest numbers, causing higher off-take and hunter effort during weekends.

Here, we use red deer (*Cervus elaphus*) to explore how weather and habitat characteristics affect the behavioral decisions of the animals and their hunters throughout the hunting season. Questions that we aim to answer are (1) Is there variation in the sex and age class of red deer harvested on farmland and in forested habitat and is this consistent with their assumed different spatial distribution and movement patterns?, (2) Do local weather conditions affect the proportion and timing of red deer harvested on farmland relative to forested habitat?, (3) Is hunter effort affected by local weather conditions and when they have the opportunity to hunt? Lastly, (4) Is the total number of red deer harvested affected by local weather conditions, hunter effort and when hunters have the opportunity to hunt? In Norway, harvest numbers of red deer have increased dramatically, from 2484 harvested individuals in 1965 and peaking at 39,070 individuals in 2010 (Statistics Norway 2012). Due to access to uniquely detailed data, the Norwegian red deer population is a useful model system to investigate how local weather and habitat types (farmland vs. forested habitat) affect hunter effort and harvesting off-take, and how this varies with age and sex class. The basis for quantifying these interactions are data on habitat type at the culling site and date of culling of harvested animals and daily data on hunter effort of red deer in Norway.

## Methods

### Study area

The data on harvested red deer cover the core area of red deer distribution in Norway, along the west coast (counties Rogaland, Sogn & Fjordane, Møre & Romsdal and Sør-Trøndelag). There are clear gradients from coast to inland and from south to north for vegetation and climatic variables; precipitation and snow depth increase from coast to inland and from south to north, while temperature decreases. The vegetation is mostly in the boreonemoral zone (Abrahamsen et al. 1977). A more detailed description of the study area can be found elsewhere (e.g., Mysterud et al. 2002, 2011). Typically, red deer are harvested both on farmland and in forested habitats in the entire study area.

### Data on harvested red deer

Hunters provided data on harvest record and hunter effort during 1995 and 1999–2010 from 11 municipalities in the counties mentioned above during the hunting season. These data derive from the “seen deer” data form that is mandatory by law in Norway when hunting cervids, and data are regarded highly reliable (Solberg and Sæther 1999; Mysterud et al. 2007). The hunting season varied between municipalities and years, but always started on September 10th and most ended on November 15th. A rutting break in the hunting season from September 26th to October 10th was present in specific municipalities during certain years. In some municipalities and years hunting season lasted until November 30th or December 23rd, but these were so few that to avoid data deficiency after this date, we excluded all harvesting statistics after November 15th. Hunters follow area-specific (set on the lowest level of the local management units) quotas based on sex and age (calves [age 6 months], yearling males [1 ½ years], adult males [2 ½ years and older], and adult females [1 ½ years and older]. All sex and age classes can be equally shot throughout the hunting season until the specific part of the quota is filled. However, a part of the quota constitutes individuals of unspecified sex and age (“optional animals”). Also, it's allowed to shoot younger animals on adult quotas. Due to these uncertainties, age- and sex-specific quotas were not available. Quotas used in the analyses to correct for availability (the percentage of quota filled) were therefore for the total number of available animals on the municipality level (available in the Table S1). Hunters noted the day of hunting, the number of harvested deer and their sex and age class (calf, yearling or older), the number of hunters participating and the number of hours spent hunting. They also noted if the individual was shot on farmland or in forested habitat. Unsuccessful hunting bouts, where no deer were harvested, were also reported in the same manner. Data were available in time series of 2–12 years from the different municipalities and included a total of 19769 harvested red deer (see Table S2 for the number of harvested animals within each habitat type, year, sex, and age class).

### Local climate variables

Daily data on temperature and precipitation were recorded by meteorological stations located within the study area and downloaded from NMI (http://www.eklima.no). If more than one station provided data within a municipality, we used the daily mean of the observations from these stations. Precipitation was available from 1 to 5 stations, and temperature was available from 1 to 2 stations within the municipalities. Preferably, we wanted to use daily data from stations within the different municipalities, but this was not always available. If a municipality lacked data on the climatic variables, we used the daily mean from the closest stations in neighboring municipalities (1–4 stations, depending on availability). Data on the moon phase were downloaded from the U.S. Naval Observatory (http://www.usno.navy.mil/USNO) as the fraction of the moon visible each day during the study period for the harvest data.

### Statistical analyses

Potential variation in the probability of red deer of different age and sex classes being harvested on farmland and the effect of weather on the probability of red deer being harvested on farmland were analyzed using generalized linear mixed-effects models for binomial data (1 = harvested on farmland, 0 = harvested outside farmland). To account for potential regional or yearly variation in harvesting, we compared models with municipality, year or both as random intercepts using Akaike's Information Criterion (AIC). Fixed effects included in the model were hunting day (1–67), mean daily temperature (°C), daily precipitation (mm; log-transformed), the fraction of the moon visible each day (continuous from 0.00 to 1.00), presence of rutting break (yes or no), age class (calf, yearling or adult), and sex of the harvested individual. We also included the proportion of yearly quota filled to correct for differences in quotas. As the mixed-effects models had problems coping with hunting day consisting of large numbers, this variable was rescaled (divided by 100) to avoid false convergence in the models. All two-way interactions with hunting day were included in the model (except for rutting break). We also included the interactions between age/sex and rutting break/prop. quota filled, and the three-way interaction between age/sex and prop. quota filled and hunting day. The interaction between moon and temperature was included as a proxy of visibility during moonlit nights, as lower temperatures during fall and winter is coupled to fewer clouds (Progulske and Duerre 1964).

Factors affecting hunter effort were investigated using generalized linear mixed-effects models for continuous data. Daily hunter effort (hours) calculated for each municipality was fitted as the response variable, and we compared models with municipality, year or both as random intercepts using AIC. Hunter effort was calculated as the number of hunters participating in a reported hunting session times the length of the hunting bout. The variable was log-transformed to assure normality. The following fixed effects were included in the full model: hunting day (1–67; rescaled), mean daily temperature (°C), daily precipitation (mm; log-transformed), the fraction of the moon visible each day (continuous from 0.00 to 1.00), day of week (categorical; weekday [Monday–Friday] or weekend [Saturday–Sunday]), and the proportion of yearly quota filled. All two-way interactions with hunting day were included, as well the interaction between moon and temperature.

Finally, factors affecting harvest numbers were investigated using generalized linear mixed-effects models for Poisson distributed data. The response variable was the number of red deer shot each day per municipality for each year, and models with municipality, year or both as random intercepts were compared using AIC. The full model included the same fixed effects as for the model investigating hunter effort: hunting day (rescaled), temperature, precipitation (log-transformed), moon fraction, day of week and prop. quota filled, as well as the variable hunter effort (the number of hunters participating in a reported hunting session times the length of the hunting bout; log-transformed). All variables except hunter effort were included in interaction with hunting day. Hunter effort was included in interactions with the remaining variables.

All mixed-effects models were fitted using the library “lme4” (Bates et al. 2014) in the statistical software R (R Core Team 2014). From the initial full models including all variables and all interaction terms, we did backwards selection based on AIC. We compared the full model to all models where one higher order interaction term was removed to identify the parameter that would yield the lowest AIC value if removed from the model. The model was refitted without the interaction term, and the process repeated until the most parsimonious model was identified.

## Results

An overview of the biological rationales investigated with corresponding results can be found in Table 1.

**Table 1:** Table showing the biological rationales investigated and our observations, with corresponding references (if available)

Biological rationale	Observation	Reference
Different spatial distribution and movement patterns will lead to variation in the sex and age class of the red deer harvest on farmland and in forested habitat	Yearlings showed largest variation in the probability of being harvested on farmland. Fig. 1A.	Ciuti et al. (2012)
	No effect of sex	Clutton-Brock et al. (1988)
	Higher probability of being harvested on farmland when there was a rutting break in the hunting season.	
Local weather conditions affect the proportion and timing of red deer harvest on farmland relative to forested habitat	Heavy precipitation = higher probability of being harvested on farmland.	Mysterud and Østbye (1999)
	Early: Higher temperature = higher probability of being harvest on farmland. Fig. 2.	Parker and Robbins (1985)
	Mid- and late-season: higher temperature = lower probability of being harvested on farmland. Fig. 2.	
	Higher probability of being harvested on farmland with increasing moonlight late in season. Fig. 1B.	
Hunter effort is affected by local weather conditions and when hunters have the opportunity to hunt	Heavy precipitation = lower hunter effort	Curtis (1971)
	Late: Higher temperature = lower hunter effort	Curtis (1971)
	Weekends = higher hunter effort. Fig. 3A.	Ciuti et al. (2012)
	Higher hunter effort with increasing moonlight late in season. Fig. 3B.	
The total number of red deer harvest is affected by local weather conditions, hunter effort and when hunters have the opportunity to hunt	Heavy precipitation = higher harvest numbers.	Mysterud and Østbye (1999)
	Mid and late: Lower temperatures = higher harvest numbers. Fig. 4A	Mysterud and Østbye (1999)
	Increased harvest numbers with increasing moonlight late in season. Fig. 4B.	
	Number harvested during weekdays and weekends depends on hunter effort. Fig. 4C,D.	Ciuti et al. (2012)
	Increasing harvest number with increasing hunter effort.	

### The effect of sex, age class, and weather on the proportion harvested on farmland

Exploring the probability of being harvested on farmland, the model with both municipality and year as random intercepts was the most parsimonious based on AIC. A summary of the final model can be found in Table 2. If not stated, all effects given in the results are predicted for mean temperature, precipitation=0, half-moon, adult deer, no rutting break, mean prop. quota filled and the first day of the hunting season.

**Table 2:** Summary of the generalized linear mixed-effects model for binomial data investigating factors affecting red deer's probability of being harvested on farmland in Norway. Municipality and year were fitted as random intercepts, with standard deviation = 0.80 and 0.95, respectively. Predictors were centered as follows: hunting day on first day of hunting season, temperature on the mean, precipitation on 0, and moon fraction on half-moon. The reference for age is adults, and no break for rutting break. N_obs_ = 19769

Variable	Estimate	SE	*z* value	*P*
Intercept	−0.883	0.364	−2.426	0.015
Temperature	0.042	0.009	4.801	<0.001
Precipitation	0.100	0.014	7.279	<0.001
Moon fraction	−0.121	0.073	−1.669	0.095
Age: Calves	0.084	0.067	1.265	0.206
Age: Yearlings	0.291	0.056	5.196	<0.001
Hunting day	−1.589	0.150	−10.587	<0.001
Rutting break: Yes	0.928	0.111	8.360	<0.001
Hunting day × Temperature	−0.249	0.022	−11.527	<0.001
Hunting day × Moon fraction	2.660	0.224	11.872	<0.001
Hunting day × Age: Calves	0.282	0.196	1.436	0.151
Hunting day × Age: Yearlings	−0.898	0.187	−4.796	<0.001
Rutting break: Yes × Age: Calves	−0.283	0.106	−2.676	0.007
Rutting break: Yes × Age: Yearlings	−0.140	0.102	−1.375	0.169

**Figure 1:**
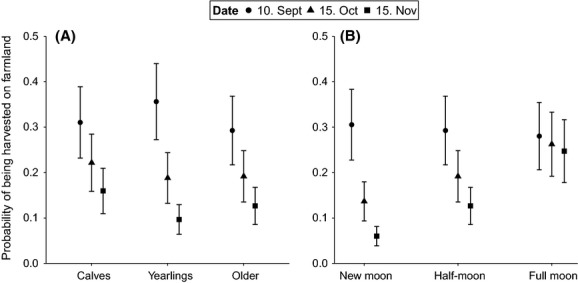
The probability of red deer being harvested on farmland through the hunting season, ± SE, for (A) different age groups and (B) different moon phases. Circles: 10. September; triangles: 15. October; quadrates: 15. November. Estimates are based on a generalized linear mixed-effects model for binomial data with municipality and year as random intercepts and N_obs_ = 19,769. The fixed effects not investigated in the plot were held as follows: temperature = mean, precipitation = 0, age = adults, moon fraction = half-moon, and rutting break = no.

The probability of being harvested on farmland decreased as the hunting season progressed (16.6% higher probability on the first day of hunting compared to the last; Table 2). This was the case for all age classes (Table 2; Fig. 1A), while yearlings showed the highest variation in probability of being harvested through the season. Yearlings had 6.4% higher probability of being harvested on farmland than adults on the first day of the season, and the pattern changed through the season, toward a reversal (Fig. 1A). Late-season, calves had the highest probability of being harvested on farmland (adults: 3.3% lower probability than calves (nonsignificant; *β* = −0.282, *P* = 0.15) and yearlings: 6.3% lower (*β* = −1.180, *P* < 0.001; Fig. 1A). The interaction between age and rutting break was also retained in the final model. All ages showed a higher probability of being harvested on farmland when there was a rutting break, than when there was no rutting break in the hunting season, and the magnitude of the increase depended on age (the difference between the two categories was larger for adults than for yearlings and calves; Table 2). Sex and the proportion of quota filled were not retained in the final model.

**Figure 2:**
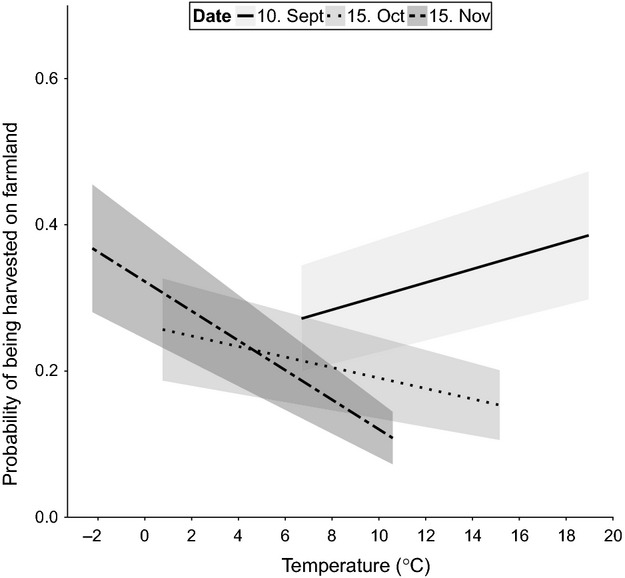
The probability of red deer being harvested on farmland through the hunting season, ± SE for different temperatures (°C). Solid line: 10. September; dotted: 15. October; dash dotted: 15. November. Estimates are based on a generalized linear mixed-effects model for binomial data with municipality and year as random intercepts and N_obs_ = 19,769. The fixed effects not investigated in the plot were held as follows: precipitation = 0, moon fraction = half-moon, age = adults, and rutting break = no.

Precipitation had a significant positive effect on the probability of being harvested on farmland (Table 2) and did not change over the hunting season (5.2% increase in the probability of being harvested on farmland with an increase from 0 to 10 mm precipitation). The effect of temperature on the probability of being harvested on farmland varied through the season (Table 2). The probability decreased with increasing temperatures mid- and late-season (0.7% and 1.5% lower probability, respectively, with 1°C increase in temperature [from 7 to 8°C]; Fig. 2). At the start of season, the relationship was positive (0.9% higher probability of being harvested with 1°C increase in temperature [from 7 to 8°C]; Fig. 2). There was a significant effect of moon phase, and this too varied through the hunting season (Table 2). The difference was largest when the moon was dark (new moon; Fig. 1B). While the probability of being harvested on farmland was high irrespective of moonlight early in the hunting season, moonlight became progressively more important in mid- and late hunting season (Fig 1B).

### Factors affecting hunter effort and the total harvest numbers

Municipality and year were retained as random intercepts in the most parsimonious models based on AIC, exploring hunter effort and the total daily harvest numbers. Model summaries with an overview of the fixed effects and interactions retained in the final models can be found in Tables 3 and 4. If not stated, all effects given in the results are predicted for mean temperature, precipitation=0, half-moon, mean prop. quota filled, mean hunter effort, weekdays, and the first day of the hunting season.

**Table 3:** Summary of the generalized linear mixed-effects model investigating factors affecting daily hunter effort of hunters harvesting red deer in Norway. Municipality and year were fitted as random intercepts, with standard deviation = 0.66 and 0.49, respectively. Predictors were centered as follows: hunting day on first day of hunting season, temperature and prop. quota filled on the mean, precipitation on 0, and moon fraction on half-moon. The reference for day of week is weekdays. N_obs_ = 3870

Variable	Estimate	SE	*t* value	*P*
Intercept	4.256	0.248	17.179	<0.001
Temperature	0.013	0.009	1.444	0.149
Precipitation	−0.052	0.013	−4.106	<0.001
Moon fraction	0.019	0.084	0.228	0.820
Day of week: Weekend	1.251	0.064	19.422	<0.001
Prop. quota filled	−1.869	0.444	−4.216	<0.001
Hunting day	−2.851	0.116	−24.520	<0.001
Hunting day × Temperature	−0.066	0.020	−3.246	0.001
Hunting day × Moon fraction	0.859	0.218	3.949	<0.001
Hunting day × Day of week: Weekend	1.292	0.169	7.655	<0.001
Hunting day × Prop. quota filled	2.424	0.828	2.929	0.003

**Table 4:** Summary of the generalized linear mixed-effects model for Poisson distributed data investigating factors affecting the daily number of red deer harvested in Norway. Municipality and year were fitted as random intercepts, with standard deviation = 0.24 and 0.12, respectively. Predictors were centered as follows: hunting day on first day of hunting season, temperature, prop. quota filled and hunter effort on the mean, precipitation on 0, and moon fraction on half-moon. The reference for day of week is weekdays. N_obs_ = 3870

Variable	Estimate	SE	*z* value	*P*
Intercept	1.308	0.085	15.400	<0.001
Temperature	−0.011	0.005	−2.110	0.035
Precipitation	0.031	0.006	5.020	<0.001
Moon fraction	0.094	0.048	1.970	0.049
Day of week: Weekend	−0.492	0.049	−10.010	<0.001
Prop. quota filled	1.078	0.220	4.900	<0.001
Hunting day	−1.186	0.078	−15.280	<0.001
Hunter effort	0.730	0.013	55.820	<0.001
Hunting day × Temperature	−0.110	0.011	−10.110	<0.001
Hunting day × Moon fraction	0.486	0.111	4.400	<0.001
Hunting day × Day of week: Weekend	0.200	0.090	2.230	0.026
Hunting day × Prop. quota filled	0.960	0.446	2.150	0.031
Hunter effort × Temperature	0.012	0.002	6.240	<0.001
Hunter effort × Moon fraction	−0.064	0.021	−3.050	0.002
Hunter effort × Day of week: Weekend	0.189	0.022	8.610	<0.001

**Figure 3:**
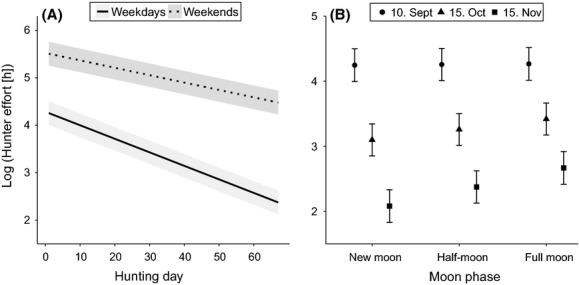
The total daily hunter effort (log of number of hunters participating in a reported hunting session times the length of the hunting bout in hours) ± SE, for (A) day of week (solid line: Monday-Friday; dotted line: Saturday-Sunday) through the hunting season and (B) moon phase (circles: 10. September; triangles: 15. October; quadrates: 15. November). Estimates are based on a generalized linear mixed-effects model with municipality and year as random intercepts and N_obs_ = 3870. The fixed effects not investigated in the individual plots were held as follows: precipitation = 0, temperature = mean, moon phase = half-moon, day of week = weekdays, prop. quota filled = mean, and hunting day = 1.

Hunter effort declined through the hunting season with 84.8% predicted fewer hours per day spent hunting at the end of season compared to the beginning (Table 3). Day of week had the strongest effect on hunter effort. Hunter effort was significantly higher during weekends, and the difference increased through the season (Fig. 3A). At the start of the hunting season, 71.4% fewer hours were spent hunting on weekdays than during weekends, and at the end of season 87.8% fewer hours were spent hunting on weekdays. Hunter effort also depended on moonlight, and the effect of moonlight changed through the season (Table 3). There was a significant effect of moonlight at the end of the season, when hunter effort increased by 79.7% during full moon periods compared to new moon (Fig. 3B). In the beginning, there was no effect of moonlight. Precipitation showed a significant negative relationship with hunter effort throughout the hunting season (Table 3). A 10 mm increase in precipitation caused a 11.6% decrease in hunter effort. The effect of temperature depended on hunting day (Table 3). There was a negative effect of temperature late in the season (3.0% decline in hunter effort with 1°C decrease in temperature), but no effect mid-season and a nonsignificant trend toward a positive effect in the beginning (1.3% increase in hunter effort with 1°C in temperature). The proportion of yearly quota filled also affected hunter effort, but differently throughout the season (Table 3). There was a negative effect of the proportion of quota filled on effort early in the season (i.e., higher proportion filled = less effort early), which disappeared toward the end. This indicates a higher proportion of the quota is filled when hunter effort is less variable through the season.

**Figure 4:**
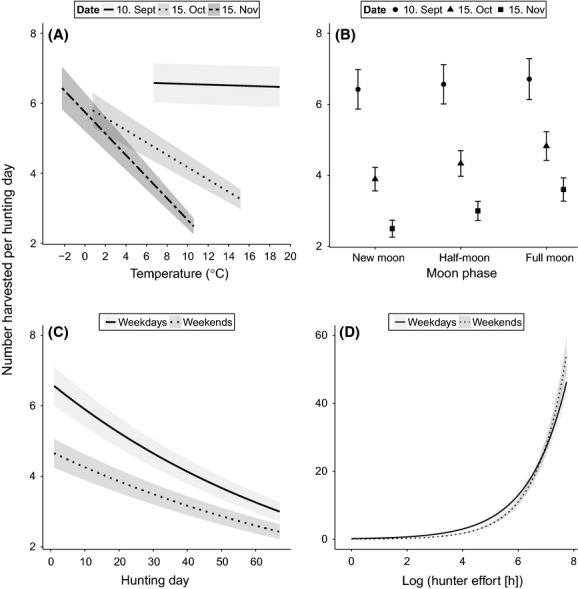
The total daily harvest numbers of red deer through the hunting season ± SE, for (A) temperature (°C; Solid line: 10. September; dotted: 15. October; dash dotted: 15. November), (B) moon phase (circles: 10. September; triangles: 15. October; quadrates: 15. November), (C) day of week (solid line: Monday-Friday; dotted line: Saturday-Sunday), and (D) hunter effort during weekdays (solid line) and weekends (dotted line). Estimates are based on a generalized linear mixed-effects model for Poisson distributed data with municipality and year as random intercepts and N_obs_= 3870. The fixed effects not investigated in the individual plots were held as follows: temperature = mean, precipitation = 0, moon fraction = half-moon, day of week = weekdays, prop. quota filled = mean, hunter effort = mean, and hunting day =1.

Overall harvest numbers declined through the season, with 54.3% fewer animals predicted harvested on the last day of the season compared to the first day (Table 4). All predictors except precipitation varied with hunting day. Precipitation was positively associated with harvest number (Table 4), with a 7.6% increase in harvest numbers when precipitation increased from 0 to 10 mm. Mid- and late-season harvest numbers decreased with increasing temperature, but there was no effect of temperature in the beginning of the season (Fig. 4A). The effect of moon phase followed the same pattern as for farmland harvesting and hunter effort (Table 4), with no effect of moonlight early in the season, and significantly higher harvest numbers with more moonlight late in the season (44.1% increase in harvest numbers during full moon as compared to new moon; Fig. 4B). The effect of day of week was large and depended on hunting day and hunter effort (Table 4). For mean hunter effort, weekend harvest numbers were always significantly lower than during weekdays (Fig. 4C). The relative difference decreased as the season progressed (41.0% lower on first day, 31.5% lower mid-season and 23.6% lower on the last day; Fig. 4C). Higher harvest numbers were predicted during weekdays than weekends when hunter effort was low to medium, while harvest numbers were highest during weekends when hunter effort was high (Fig. 4D). The effect of hunter effort on harvest numbers also varied with temperature and moonlight (Table 4). Overall, there was a strong positive effect of hunter effort on total harvest numbers, but the relative increase in harvest numbers declined with increased hunter effort (i.e., harvest numbers increased 65.9% from 20 to 40 h hunter effort, but only 34.4% from 40 to 60 h). The proportion of quota filled was positively associated with harvest numbers throughout the season (Table 4). Higher harvest rate with higher proportion of quota filled both early and late in the season indicates that quota is not a limiting factor for harvest in most areas.

## Discussion

Despite the large number of studies on how selective harvesting and climate affect deer populations, there are very few studies linking how prevailing weather may affect harvesting indirectly either through age- and sex-specific animal behavior (use of farmland) or hunter behavior (effort). We found a small effect of age on the probability of being harvested on farmland, with calves having a higher probability of being harvested on farmland late in the season, and a nonsignificant trend toward younger animals (yearlings) having a higher probability of being shot on farmland early in the season, probably due to the lack of experience of younger animals. We found no effect of sex. Precipitation and temperature had a variable influence on the probability of being harvested on farmland and on overall harvest numbers. Although weather played a measurable role, the effect of moon phase was a stronger predictor of the probability of being harvested on farmland, and together with day of week, on overall harvest numbers and hunter effort.

Our results show that making predictions about harvest numbers and probabilities based on changing weather conditions and habitat choice is not straightforward. The relationship between animal behavior (the probability of being harvested on farmland) and the prevailing weather varied throughout the season, that is, with low temperatures not always increasing the probability of being harvested in open habitats. Hunter effort seemed to have a more consistent response to weather variables, moon phase and day of week from the beginning to the end of the season (i.e., always higher hunter effort during weekends and with decreasing precipitation). Our results show that the interaction effects of prevailing weather and space use on animal and hunter behavior are complicated and may be less important than hunter preference (selective shooting) and quotas in determining patterns of hunter selection and harvest off-take.

### Do different age and sex classes experience different risks of being harvested?

Ciuti et al. (2012) found that female elk decreased their movement rate and avoided open areas more often with increasing age, suggesting a learning effect as the animals age. In moose, hunters overestimated the population of males during hunting season, as males exposed themselves more often to the hunters (Solberg et al. 2010). Young, inexperienced males should therefore have a higher probability of being harvested in open habitats like farmland. In the monomorphic red grouse (*Lagopus lagopus scoticus*), sex- and age-specific behavioral differences caused differential vulnerability to harvesting and a larger off-take of young animals at large bag sizes (Bunnefeld et al. 2009). We did not find any differences between the sexes, but calves had the highest probability of being harvested on farmland late in the season, and there was a tendency for the same pattern for yearlings early in the season. Toward the end, yearlings had the lowest probability of being harvested on farmland, perhaps indicating that learning is already taking place, or the pattern can arise due to depletion of yearlings. Further, we cannot ignore effects of how hunter preferences change during the season. Hunters often seek to harvest the yearling quota early in the hunting season, as they are easier to separate from older (and younger) animals then. Also, hunters are expected to be less choosy toward the end of the hunting season in order to fill their quotas. This behavior might yield unintentional harvesting selection, as is the case for red grouse (Bunnefeld et al. 2009). Bunnefeld et al. (2011) demonstrated that in red grouse unintentional selection of specific age or sex classes can lead to decreased population yield at high harvesting rates.

The presence of a rutting break in the harvest season increased the probability of being harvested on farmland for all age groups. The magnitude of the increase depended on age, with adults experiencing a larger difference than yearlings and calves between seasons with and without a rutting break. Many species are known to shift their habitat use into safer habitats in response to human disturbance (review in Frid and Dill 2002). Lowered human activity during the rutting break could therefore lead the deer to increase their use of open, risky habitats. A higher number of animals may then be available on farmland when the hunting resumes after the break, thus increasing the harvesting probability in the habitat.

### Fall weather conditions, harvest numbers, and hunter effort

The relationship between fall weather conditions, habitat, and harvesting risk varied through the hunting season. Habitat characteristics have been shown to affect predation risk in ungulates and success for their predators (e.g., moose and wolf; Kunkel and Pletscher 2000). Local weather is experienced very differently both by hunters and their prey in open and closed habitats (Curtis 1971; Mysterud and Østbye 1999) and is therefore also expected to interact with habitat when determining the probability of being shot. Precipitation was the most consistent weather variable for all analyses, as it did not change over the season. However, precipitation affected animal and hunter behavior differently. Precipitation showed a negative relationship with hunter effort throughout the hunting season and affected harvest numbers and the probability of being harvested on farmland positively. Increased precipitation is known to cause increased heat loss in ungulates (Barrett 1981; Parker 1988), and a response is to seek cover when precipitation is heavy (Mysterud and Østbye 1999). Hunters are expected to find less pleasure in hunting during heavy rain, also possibly with fewer animals exposing themselves, and we found hunter effort to be consistently lower during days of heavy precipitation. The probability of being harvested on farmland instead of other habitats showed a positive relationship with precipitation. We expected cervids to hide in covered habitats during heavy precipitation (Mysterud and Østbye 1999), so this observation could reflect that deer in general are less sensitive to prevailing weather when selecting foraging locations (Moen 1976). Deer are also known to emerge into open habitats once the precipitation stops (pers. obs.). This could cause the lack of a clear effect of precipitation on harvesting probability, as this event would not be caught by our analysis using daily precipitation measurements. The positive relationship between precipitation and harvest numbers could be due to precipitation being more likely to be falling as snow late in the season, which is less effective in wetting of the pelage. Thus heat loss will be lowered (Mejdell and Boe 2005), causing a lack of response in the daily harvest numbers.

The effect of temperature varied through the hunting season for both animal and hunter behavior. Mid- and late-season, the probability of being harvested on farmland instead of forest decreased with increasing temperatures. When they have a choice, red deer spend most of their time in covered habitats, especially if these provide both forage and shelter, and open forage-rich habitats are used only as much as needed to cover their energy requirements (Godvik et al. 2009). During the hunting season, open farmland provides forage of higher quality compared to forested habitat (Albon and Langvatn 1992). When it is colder, ungulates may have higher energy requirements (Parker and Robbins 1985) and can therefore benefit from foraging on the high-quality forage found in open habitats, increasing the probability of being harvested due to exposure. In fall and winter low temperatures after dark are also coupled to few clouds and less precipitation (Progulske and Duerre 1964), which could mean higher visibility during moonlit nights and thus higher risk of red deer being shot on farmland. Early in the season, the probability of being harvested on farmland increased with increasing temperature. Cervids are known to spend more time in covered habitats during cold periods to avoid heat loss (Mysterud and Østbye 1999), which could explain the relationship found early in the hunting season.

For hunter effort and total harvest numbers, we found the same negative relationship with temperature as for the probability of being harvested on farmland mid- and late-season, but the effect was somewhat weaker for hunter effort. Extreme cold is likely to discourage most hunters from spending time outside (Curtis 1971). The lack of a positive response from hunters could be due to very low temperatures being rare, which may mean that the weather was not sufficiently cold for the hunters to react and show a response of decreased effort. For the red deer, the negative relationship with temperature could arise when increased energy requirements during cold weather (Parker and Robbins 1985) force the animals to use more farmland and thus expose themselves for the hunters, as found above (higher probability of being harvested on farmland during cold days).

### Other factors affecting harvest numbers and hunter effort

When clouds are few, a full moon provides more light and increased visibility (Janiczek and DeYoung 1987), for both hunters and for prey. For predator–prey relationships, an increased visible fraction of the moon has been shown to increase hunting success and activity for certain predators (wolves; Theuerkauf et al. 2003; cheetah (*Acinonyx jubatus*) and African wild dog (*Lycaon pictus*); Cozzi et al. 2012). Human hunters are also known to make use of the extra light and increase hunter effort during full moon periods, particularly on farmland, as we found in our study system. We also found an increase in the probability of being harvested on farmland and in harvest numbers during full moon, but the effect was apparent only mid- and late-season. It is likely that the decrease in periods of daylight, causing longer nights as the hunting season progresses on the Northern hemisphere, makes moonlight hunting more attractive to hunters, and thereby increasing harvesting risk.

Off-road human activity is higher during weekends (Ciuti et al. 2012). Our hunters showed a marked increase in hunter effort during weekends as compared to weekdays, and this was reflected in increased harvest numbers during weekends when hunter effort was high. While weekend hunter effort was always higher than during weekdays, harvest numbers through the week varied with hunter effort. Low to medium hunter effort yielded higher harvest numbers during weekdays, while high hunter effort yielded higher harvest numbers during weekends. However, as the relative difference between weekday and weekend hunter effort increased through the season, the relative difference for harvest numbers did not. Human disturbance is known to affect movement rates and cause displacement of wild animals (Frid and Dill 2002; Ciuti et al. 2012). As there is a learning effect in how ungulates respond to humans (Geist 1971), the lack of a corresponding increase in harvest numbers in spite of increased effort could reflect a learning effect in the red deer, as found in other ungulates (e.g., sheep [Ovis spp]; Dwyer 2004). It may, however, be more likely that the discrepancy between harvest numbers and hunter effort during weekends reflects a depletion of accessible animals and/or filled quotas toward the end of the harvest season, or that hunters who continue hunting on weekdays often are more experienced and efficient than hunters who hunt mainly on weekends. The effect of hunter effort on total harvest numbers was also dependent on temperature and moon phase, again showing how many factors interplay in determining patterns in harvesting. Clearly, we are only starting to grasp the interactions between weather and animal and hunter behavior, but our study suggests that these issues enable a further understanding of the intricate interactions in harvested deer populations.

### Future perspectives

Key components often recognized when considering sustainability in harvested systems are the life history of the species and the management objectives, while hunter behavior is often ignored when formulating guidelines (Arlinghaus et al. 2008; Johnston et al. 2010). The importance of including behavior for understanding human harvesting has been acknowledged as an important basis to achieve sustainable management of recreational fisheries (Johnston et al. 2010, 2013; Hunt et al. 2011). This subject is likely to become increasingly important also in deer management, now that deer numbers are increasing in Europe while the number of hunters is decreasing (Andersen et al. 2014). Populations of hunters consist of different types, each with different goals and preferences, that is, some hunting mainly for trophies, others for meat, for population control and more. Groups of hunters with different motivations can differ largely in their effectiveness (Andersen et al. 2014), and hunter behavior through different methods (Martinez et al. 2005; Torres-Porras et al. 2009) or categories of hunters (Mysterud et al. 2006; Rivrud et al. 2013) can influence the composition of the harvest. Knowledge about heterogeneity and dynamics among hunters and their corresponding variation in hunter preference should therefore be incorporated when formulating management rules. Such insight can be used in order to influence hunter behavior in such a way as to change the sex ratio and/or age distribution of the harvested populations, for example through different price categories or monitoring of hunter behavior which can be implemented into management guidelines. Future studies should therefore seek to understand the dynamics of hunter behavior, how this can be influenced, and utilize this in the interplay between hunters, animal life histories and management rules to obtain sustainable off-takes in managed populations.

## Supporting Information

**Table S1.** Table showing the proportion of the yearly harvest quota filled for each municipality. **Table S2.** Table showing the number of red deer observations per habitat type, sex, age and year used in the analyses.
