# Supplementary material for: Interaction effects between weather and space use on harvesting effort and patterns in red deer
Source: Ecol Evol. 2014 Dec 3;4(24):4786–97. doi: 10.1002/ece3.1318 (PMC4278827; doi:10.1002/ece3.1318)
Supplement: Supplementary file 1 [file ece30004-4786-sd1.docx]

# Supporting Information

**Table S1.** Overview of the proportion of quotas filled for each municipality (columns) and year (rows).

| **Year** | **Agdenes** | **Bremanger** | **Eid** | **Flora** | **Gloppen** | **Hornindal** | **Kvinnherad** | **Meldal** | **Orkdal** | **Stryn** | **Tingvoll** |
| --- | --- | --- | --- | --- | --- | --- | --- | --- | --- | --- | --- |
| 1995 | - | - | - | - | - | - | 0.75 | - | - | - | - |
| 1999 | - | - | - | - | - | - | 0.85 | - | - | 0.86 | 0.68 |
| 2000 | - | - | - | - | - | - | 0.80 | - | - | - | 0.77 |
| 2001 | - | 0.71 | - | - | - | - | 0.75 | - | - | 0.80 | 0.71 |
| 2002 | - | 0.74 | - | - | - | - | - | - | - | - | 0.74 |
| 2003 | - | 0.82 | - | 0.82 | 0.69 | - | 0.78 | - | - | - | 0.80 |
| 2004 | 0.65 | 0.78 | - | 0.82 | 0.63 | - | 0.76 | - | - | - | 0.82 |
| 2005 | 0.64 | 0.83 | 0.85 | 0.82 | 0.56 | 0.73 | 0.76 | - | 0.68 | 0.79 | 0.82 |
| 2006 | 0.54 | 0.76 | 0.73 | 0.85 | 0.65 | 0.84 | 0.77 | - | - | 0.85 | 0.85 |
| 2007 | 0.87 | 0.88 | 0.85 | 0.89 | 0.74 | 0.94 | 0.87 | 0.60 | 0.75 | 0.99 | 0.90 |
| 2008 | 0.72 | - | 0.86 | 0.87 | 0.63 | 0.71 | 0.86 | 0.58 | 0.81 | 0.91 | 0.88 |
| 2009 | - | - | - | - | - | - | - | - | - | - | 0.84 |
| 2010 | - | - | - | - | - | - | - | - | - | - | 0.90 |

**Table S2.** Overview of the number of harvested red deer used in analyses divided by habitat type, year, sex and age class. N_obs_ = 19769.

|  | **Farmland** | | | | | | **Outside farmland** | | | | | |
| --- | --- | --- | --- | --- | --- | --- | --- | --- | --- | --- | --- | --- |
|  | **Male** | | | **Female** | | | **Male** | | | **Female** | | |
| **Year** | **Calf** | **Yearling** | **Older** | **Calf** | **Yearling** | **Older** | **Calf** | **Yearling** | **Older** | **Calf** | **Yearling** | **Older** |
| 1995 | 1 | 0 | 1 | 0 | 0 | 0 | 14 | 29 | 53 | 8 | 18 | 39 |
| 1999 | 30 | 40 | 60 | 22 | 19 | 51 | 55 | 73 | 100 | 38 | 32 | 91 |
| 2000 | 36 | 47 | 46 | 26 | 24 | 67 | 46 | 81 | 83 | 32 | 37 | 95 |
| 2001 | 33 | 64 | 54 | 36 | 18 | 58 | 71 | 89 | 98 | 37 | 53 | 102 |
| 2002 | 9 | 3 | 10 | 15 | 20 | 22 | 32 | 51 | 83 | 28 | 52 | 63 |
| 2003 | 56 | 60 | 87 | 44 | 78 | 104 | 109 | 161 | 225 | 108 | 131 | 230 |
| 2004 | 77 | 88 | 85 | 58 | 68 | 131 | 130 | 183 | 214 | 117 | 144 | 271 |
| 2005 | 122 | 209 | 185 | 91 | 164 | 204 | 262 | 373 | 518 | 222 | 351 | 501 |
| 2006 | 129 | 243 | 234 | 130 | 144 | 249 | 247 | 416 | 538 | 219 | 346 | 483 |
| 2007 | 192 | 268 | 283 | 148 | 220 | 287 | 298 | 430 | 559 | 255 | 364 | 526 |
| 2008 | 162 | 248 | 227 | 138 | 187 | 231 | 266 | 356 | 464 | 201 | 316 | 483 |
| 2009 | 24 | 22 | 26 | 25 | 33 | 46 | 34 | 20 | 28 | 34 | 37 | 64 |
| 2010 | 19 | 12 | 19 | 21 | 23 | 27 | 25 | 24 | 18 | 12 | 12 | 21 |
